# The tale of two talins – two isoforms to fine‐tune integrin signalling

**DOI:** 10.1002/1873-3468.13081

**Published:** 2018-05-18

**Authors:** Rosemarie E. Gough, Benjamin T. Goult

**Affiliations:** ^1^ School of Biosciences University of Kent Canterbury UK

**Keywords:** integrin, mechanobiology, talin

## Abstract

Talins are cytoplasmic adapter proteins essential for integrin‐mediated cell adhesion to the extracellular matrix. Talins control the activation state of integrins, link integrins to cytoskeletal actin, recruit numerous signalling molecules that mediate integrin signalling and coordinate recruitment of microtubules to adhesion sites via interaction with KANK (kidney ankyrin repeat‐containing) proteins. Vertebrates have two talin genes, *TLN1* and *TLN2*. Although talin1 and talin2 share 76% protein sequence identity (88% similarity), they are not functionally redundant, and the differences between the two isoforms are not fully understood. In this Review, we focus on the similarities and differences between the two talins in terms of structure, biochemistry and function, which hint at subtle differences in fine‐tuning adhesion signalling.

## Abbreviations

**ABS**, actin‐binding site

**DD**, dimerisation domain

**ECM**, extracellular matrix

**FA**, focal adhesion

**FERM**, 4.1 protein, ezrin, radixin, moesin

**IBS**, integrin‐binding site

**IF**, intermediate filament

**PTM**, post‐translational modification

**VBS**, vinculin‐binding site

## Integrin adhesions: linking the cell to the extracellular matrix

Integrin‐mediated adhesions to the extracellular matrix (ECM) are found in nearly all cell types and mediate a diverse range of functions. There are 24 αβ heterodimeric integrins, which show distinct patterns of cell‐type and tissue‐specific expression, and support different forms of cell‐ECM and cell‐cell attachment. Integrins connect to the ECM via their large extracellular domains but, in contrast, the cytoplasmic domains, the ‘integrin tails’, are generally short (~ 40–60 amino acids). Despite this diminutive size, large multiprotein complexes assemble on the cytoplasmic face of integrins, providing linkages to the cell cytoskeleton and to numerous intracellular signalling pathways.

The complexity of cell‐matrix adhesions has been highlighted by the analysis of the ‘integrin adhesome’ using mass spectrometry on multiple integrin adhesion complexes. This identified a network of > 240 proteins 1, 2, and additional adhesome proteins are constantly being discovered, many of which are cytoplasmic components that couple adhesions to numerous signalling cascades. These enable diverse intracellular responses, a process often referred to as ‘outside‐in’ signalling. These signalling hubs regulate a multitude of cellular processes including cytoskeletal dynamics and cell motility, cell growth, survival and the cellular response to the local environment. Unsurprisingly, numerous diseases arise from the defects in components of the integrin adhesome 3. Further analysis of integrin adhesome datasets collected under different conditions has revealed the dynamic nature of these complexes, and the functional diversity that can derive from the same building blocks. What emerges is a consensus adhesome of ~ 60 proteins centred around four axes comprising; ILK‐PINCH‐kindlin, FAK‐paxillin, talin‐vinculin and α‐actinin‐zyxin‐VASP, although it seems likely that all of these axes are linked to talin in some way. As well as 24 different integrins, vertebrates also have two major talin isoforms: talin1 and talin2.

Most of the attention on talin has focused on talin1, primarily due to its essential role in mediating cell adhesion as shown by studies on talin1 knockout 4 and talin1‐depleted cells 5, 6, 7. Talin1 knockout is also embryonic lethal in mice due to arrested gastrulation, indicating a key role in early development 4. In contrast talin2, which was only discovered following publication of the human genome sequence 8, 9 has received less attention, and the fact that talin2 knockout mice are viable and fertile 10 suggests isoform redundancy. However, talin2 knockout mice display a mild dystrophic phenotype and the variability in the number of pups surviving to adulthood suggest underlying defects 10. Therefore, it appears that talin2 plays important roles in development although many of its functions can be compensated for by talin1.

This Review aims to summarise what is currently known about the structural, biochemical and functional differences between the two talin isoforms. Evolutionary genomics has been used to study talin isoforms in the past, and here, we combine genetic analysis with the recent wealth of structural information to highlight the emerging functions of the two talins as signalling platforms.

## Integrin–talin–actin: the core of cell‐matrix adhesions

**Figure 1:**
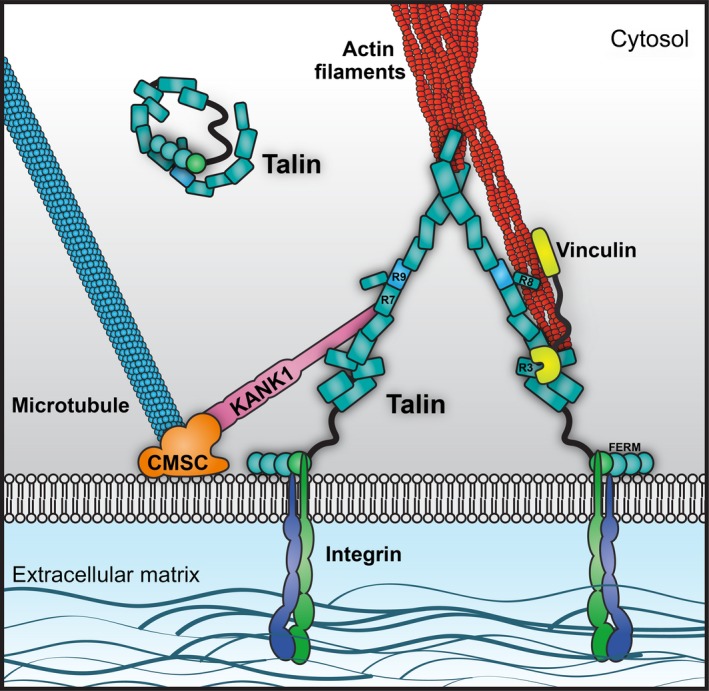
Talin at the core of the adhesion. A cartoon of the core of integrin adhesions, highlighting talins central role. Talin coordinates both the actin cytoskeleton, and through the interaction with KANK proteins, the microtubule cytoskeleton at adhesion sites. Once the adhesion core is assembled, talin serves as a scaffold to recruit many other proteins in order to form all the many different types of adhesive structures (focal adhesions, podosomes, invadopodia, etc.).

Strikingly, despite the structural complexity of cell‐ECM adhesions, the majority of these dynamic adhesion complexes comprise a simple and robust core of three proteins: talin which binds to and activates integrins and couples them to the actin cytoskeleton (Fig. 1). All of the other components can be assembled on to this framework to give rise to various types of adhesive structures. Once formed, the protein vinculin is recruited to the complex to stabilise the connection to actin, a process which is regulated by an elegant feedback mechanism. Thus, vinculin is only recruited when talin experiences mechanical force, and force will only be exerted when talin is successfully bound to an integrin *and* coupled to actin. If these conditions are not met, then nascent adhesions will not experience sufficient force to recruit vinculin and will disassemble. However, once vinculin is recruited to nascent adhesions, it crosslinks talin to actin, and the core, linkages are stabilised. How such complexes mature depends on multiple variables including cell type, ECM composition, matrix stiffness, integrin subtype, mechanical signals, etc. This leads to the development of a variety of adhesion complexes including nascent adhesions, focal adhesions (FAs), fibrillar adhesions, podosomes, invadopodia, etc., all of which have at their core the same integrin–talin–actin connection. In their recent Review, Klapholz and Brown eloquently describe the myriad of different roles that talin plays in adhesion, and provocatively call talin ‘the master of integrin adhesions’, a view we share 11.

## The talins

Talin is a large 270 kDa actin‐binding protein that was first discovered in 1983 as a component of FAs and ruffling membranes 12. Talin comprises an N‐terminal FERM domain (the head) coupled to a flexible talin rod. Since then, it has been shown to be a key component of integrin adhesions with roles in integrin activation 13, the molecular clutch that couples integrins to cytoskeletal actin 14, FA assembly and the recruitment of numerous signalling molecules 15. Talins also interact with the KANK family of adapter proteins 16, 17 which target microtubules to adhesion sites, stimulating FA turnover 18. As well as transmitting forces between integrins and the actin cytoskeleton, the length of talin has been shown to define the geometry of the adhesion 19, and talin plays a key role as a mechanosensitive adapter, undergoing force‐dependent conformational transitions in its 13 rod domains 20, 21, 22, 23, 24 that modulate binding interactions with mechanosensitive ligands. Given the above, it is unsurprising that talin1 knockout in mice is embryonic lethal 4, 25.

Tissue expression and cellular localisation vary considerably between the two isoforms; talin1 is expressed in all tissues. In contrast, talin2 expression is more variable, and it is absent entirely from some cell types, for example, no talin2 is present in endothelial cells possibly via silencing of the *Tln2* gene by promoter methylation 26, 27. The Human Protein Atlas 28 shows the near ubiquitous expression of talin1 in all cell types in all tissues, whereas the high levels of talin2 are found mainly in the brain, particularly the cerebral cortex, heart muscle and the kidney.

There is clear interplay in the expression of the two talin isoforms, although the mechanism for this is not known. Talin2 expression is rapidly upregulated following knockout of talin1, both transiently 26 and also in *Tln1*‐knockout mouse embryonic fibroblasts 7 leading to rescue of many of the consequences of the loss of talin1. However, knockout of both talin isoforms completely ablates cell‐ECM adhesion 5, confirming the essential role of talins in integrin biology.

In fibroblasts, both talins localise to FAs, and talin1 is recruited directly to the leading edge, via proteins like the Rap1 effector RIAM (Rap1‐interacting adapter molecule) 29, 30 and FAK (focal adhesion kinase) 31. In contrast, less is known about talin2 recruitment. Although talin2 binds to RIAM, talin2‐specific antibodies reveal that talin2 forms diffuse aggregates throughout the cell, which overtime coalesce to form larger complexes, either at focal or fibrillar adhesion sites 25. For the most part, only talin2 is found at fibrillar adhesions in the centre of the cell 25. This localisation positions talin2 at sites of fibronectin secretion and assembly 32 and also to formation of invadopodia 33. Although much less is known about the role of talin2, it has recently been the subject of increased interest and isoform‐specific functionalities have been reported. For example, talin2 has been reported to be indispensable for the generation of traction force and invadopodium‐mediated matrix degradation required for invadopodia formation 33. Furthermore, talin2 has been shown to be able to recruit vinculin in the absence of mechanical force suggesting different mechanical properties 34. In summary, the relative roles of talin1 and talin2 remain to be fully elucidated.

## Structure of talin 1 and 2

### Gene structure and splice variation in talins

The two talins are encoded by separate genes, *Tln1* and *Tln2*, which have conserved intron–exon boundaries 9, 35. However, whereas talin1 has relatively small introns resulting in a gene of ~ 30 kb, talin2 is much bigger (~ 190 kb), due to the presence of much larger introns. Moreover, initial studies suggest that multiple talin2 isoforms are generated via differential splicing 36. While the function of these isoforms is currently unknown, the expression pattern of each is distinct. Testes, kidney and brain express short C‐terminal proteins lacking the FERM domain 36 raising the possibility that such variants might function independent of integrins, although they do contain the integrin‐binding site located in the rod domain. Intriguingly, expression of a C‐terminal talin1 fragment resembling the testes‐specific talin2 isoform was sufficient to rescue cell cycle progression in talin1‐depleted cells suggesting a role in cellular signalling 37, 38.

The ancestral *Tln* gene appears to have undergone duplication in chordates with the emergence of vertebrates to give rise to talin1 and talin2 39. Invertebrates and simple chordates have a single talin gene; vertebrates have two. Chordates can be divided into three major groups: Craniata (including the vertebrates), Cephalochordata (including the lancelets) and Tunicata (including sea squirts). Since the original publication on talin evolution 39, the genomes of *Petromyzon marinus* (one of the Cyclostomata, a jawless vertebrate) 40 and Branchiostoma floridae (a lancelet, one of the Cephalochordata) 41 have been published. Strikingly, the Branchiostoma has only a single talin, whereas the Petromyzon genome encodes two. This confirms and extends the original conclusions about talin evolution 9, 39 and suggests that the genome duplication leading to present day talin1 and talin2 took place before the divergence of jawed and jawless vertebrates, but after (or with) the divergence of the craniates from other chordates. The acquisition of two different copies of talin appears to be beneficial to an organism; talin gene duplication has also occurred in Amboebozoa and in *Dictyostelium discoideum*. The Dictyostelium talin genes, *TalA* and *TalB*, encode proteins with distinct functions, with TalA required for cell–substrate adhesion, phagocytosis and cytokinesis, and TalB required for the force transmission required to support morphogenetic movements during differentiation 42.

### Talin domain structure

Remarkably, despite millions of years of evolutionary time since talin first appeared and since the two talins diverged, the length of both the major talin isoforms has remained almost identical (talin1: 2541aa; talin2: 2540aa). Furthermore, both major isoforms have identical domain structure and contain 18 domains. This invariability is in stark contrast to many other multidomain proteins such as titin, spectrin and filamin – these have varied in length, increasing and decreasing in size through evolution until reaching the length we see today 43, 44, 45. This unvarying domain arrangement in all available talin sequences suggests that each domain has a role that is universally required (it is worth mentioning that in some nonvertebrate organisms, including Dictyostelium and Drosophila, talin has acquired additional C‐terminal residues that extend beyond the universal domain arrangement 39, 46). The following discussion of talin domain structure, therefore, applies to both talin1 and talin2.

#### The talin head

Talins consists of an atypical N‐terminal FERM (4.1 protein, ezrin, radixin, moesin) domain, known as the talin head containing four subdomains F0–F3 47, 48 rather than the three subdomains (F1–F3) found in most other FERM domain proteins. Moreover, the crystal structure of the talin1 FERM domain shows a linear domain structure 47 rather than the cloverleaf structure found in other FERM domain proteins. The structure of the talin2 FERM domain confirms this linear domain arrangement (our unpublished data). Extensive studies show that it is the talin F3 subdomain that directly engages the beta‐integrin cytoplasmic tail via the first (membrane proximal) of two NPxY motifs in the tail 49. The integrin‐binding interfaces have been characterised in both the talin1 and talin2 F3 subdomains, and this has revealed that conserved residue changes in the binding surfaces tune the affinities of the two talins for different integrin tails 50. For example, the ubiquitous beta1a‐integrin was recently shown to bind preferentially to talin2 51 whereas the muscle‐specific beta1d‐integrin has a threefold higher preference for talin2 over talin1 33, 52, 53. This provides selectivity for different talin and integrin complexes, and different couplings are likely to regulate different cellular functions 54.

However, whilst F3 is the only talin head subdomain that engages the integrin, F3 in isolation is not very effective at activating integrins and the other head subdomains are also required to make an effective ‘integrin activation lock’ and maintain the integrin in the active, high‐affinity conformation 55. The other head subdomains achieve this by interaction with phosphoinositides such as PtdIns(4,5)P_2_ (PIP2) in the plasma membrane; a basic surface on the F2 subdomain mediates interaction with the plasma membrane, which applies torque on the integrin to stabilise the active conformation 52, 56, 57. In addition, the F1 subdomain contains a large (~ 30aa) unstructured insertion, the F1‐loop, which, via a cluster of positively charged residues, interacts with PIP2 and is essential for integrin activation 58. The F0 subdomain has been shown to bind the membrane‐tethered small GTPase, Rap1 58, 59, 60 and this interaction has been implicated in membrane targeting of talin to the plasma membrane 59. Interestingly, the additional F0 subdomain and the F1‐loop elements of the talin FERM domain are also found in the kindlin family of proteins 58, 61 which synergise with talin to activate integrins 62. These features are not found in other FERM domain proteins and are unique to integrin‐activating FERM domain proteins.

As well as binding to integrins, Rap1 and the membrane, the talin head (via the F3 subdomain) has been shown to bind to PIP kinase gamma 63, which is thought to generate the PIP2 required to support integrin activation 64. Beyond this, the F3 subdomain has emerged as showing remarkable ligand‐binding plasticity and has been linked to binding FAK 31, TIAM1 (T‐cell lymphoma invasion and metastasis 1) 65, layilin 66, Gα13 (G‐protein subunit *Galpha13*) and RIAM 67 all via the same site. The hierarchy of these interactions, that are presumably mutually exclusive with integrin binding and each other, is not yet fully understood. Talin contains three actin‐binding sites (ABS1‐3) 68. ABS1 is in F2‐F3 in the talin head 69 and has recently been shown to be important for capping actin filaments to block actin polymerisation 70.

#### The talin rod

**Figure 2:**
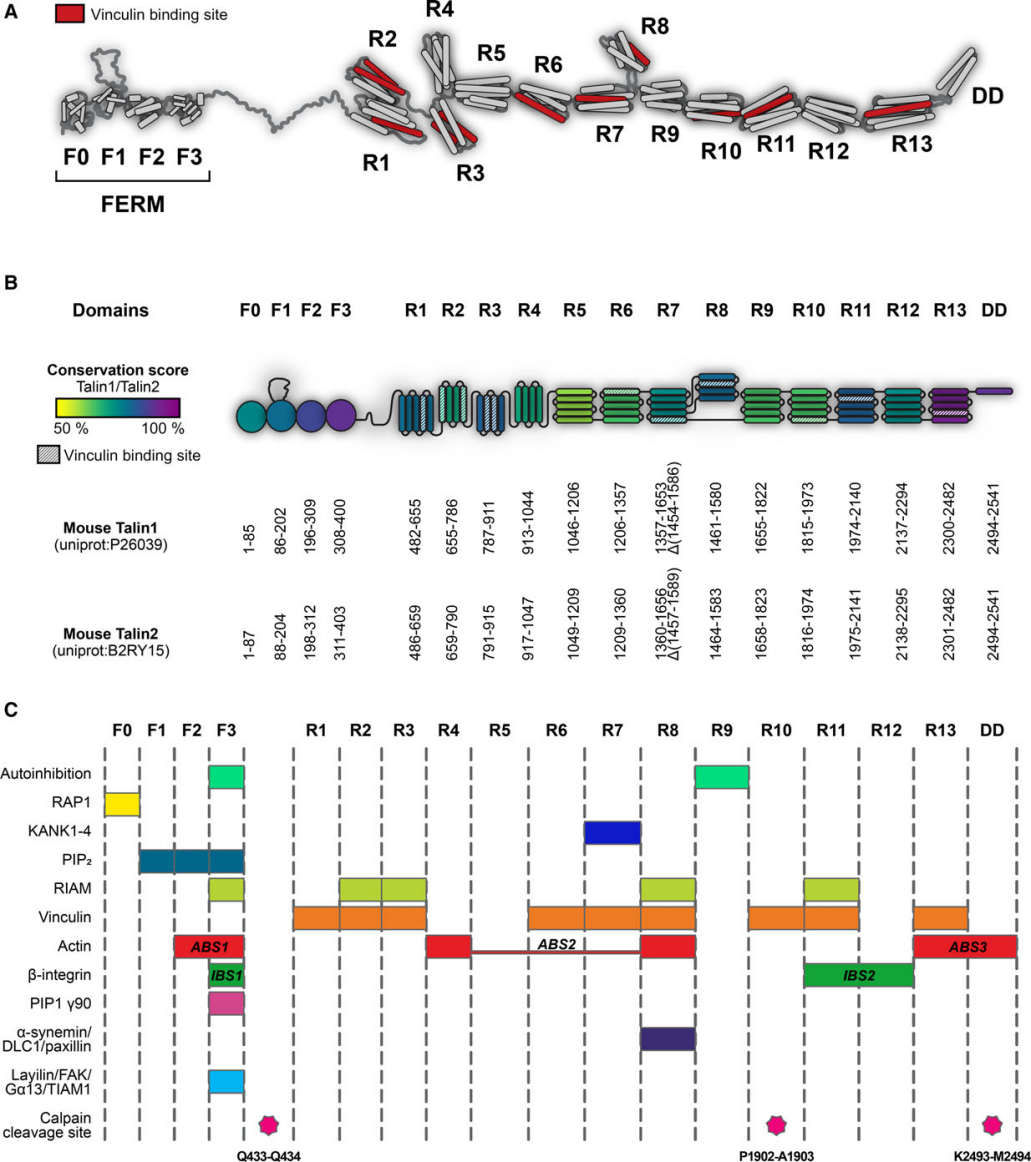
Structure and domain map of the two talin isoforms. (A) Structural model of talin showing the domain arrangement of talin. Vinculin‐binding sites are shown in red. The N‐terminal talin head comprising F0–F3 and the talin rod domains R1–R13 are shown. (B) Schematic representation of the talin domain structures coloured by sequence identity between the two isoforms. The domain boundaries are given for mouse talin1 (UniProt: http://www.uniprot.org/uniprot/P26039) and talin2 (UniProt: http://www.uniprot.org/uniprot/B2RY15). Provided that these boundaries are used, it is possible to make any talin fragment or delete any talin domain while maintaining the structural integrity of the protein. (C) The locations of many of the talin ligand‐binding sites are shown, as are the calpain cleavage sites.

The talin head is connected, via an 82‐amino acid unstructured 71 calpain‐sensitive linker 72, 73 to the large 2000 residue talin rod that is made up of 62 α‐helices. We have recently determined the boundaries and structures of the talin1 rod domains showing it contains 13 domains (R1–R13) 24 organised into two functionally distinct regions, a linear C‐terminal rod‐like region comprised of 5‐helix bundles and a compact N‐terminal region where three 4‐helix bundles (R2–R4) are inserted into the series of 5‐helix bundles (Fig. 2b).

Structural analysis of the talin rod was complicated as only two regions of the rod have sequence homology to other proteins: R13, which contains an I/LWEQ domain 8, 74, and the central region of the rod (resolved to be R7–R8) which has homology to a protein of unknown function, MESDC1 75. The rest of the rod lacks homology to other proteins meaning prediction of the domain boundaries *a priori* was not possible. Part of the reason for the lack of homology of the talin rod to other proteins turned out to be that 8 of the 13 talin rod domains – R1, R5–R7, R9–R12 – contain a 5‐helix bundle fold, the ‘talin rod fold’, that has so far only been recognised in talin.

#### The talin rod fold

Although 4‐helix bundles are common in nature (the 4‐helix up–down bundle present in R2, R3, R4 and R8 is a common fold (SCOP 47161 76)), 5‐helix bundles are unusual. At the core of the talin rod fold is a common 4‐helix up–down, left‐handed twist topology as seen in numerous 4‐helix bundles. However, in the talin rod, this fold is augmented by an extra N‐terminal helix, connected by a long (~ 9 residue) loop that allows the first helix to pack against helices 3 and 4 of the bundle (Fig. 2) to form a 5‐helix bundle. This addition of an extra helix to the talin rod domains has profound effects on talin function, not least because 5‐helix bundles, where the N‐ and C‐termini are located at opposite ends of the bundle are optimal for forming a rod‐like arrangement (Fig. 2). The linear rod‐like region is perfectly designed to transmit forces, which act on the compact N‐terminal region. Furthermore, the additional helix significantly enhances the thermal and mechanical stability of the domains 22, 77, helping provide different mechanical responses for each domain 21 and restricting access to the cryptic vinculin‐binding sites (VBS) buried inside many of the domains. As a result, each rod domain has its own unique properties, and this is central to talins role as a mechanosensor (see next section). Unfolding 5‐helix bundles by pulling on the termini positioned at opposite ends of the bundle is restricted by extensive contacts throughout the length of the helices and requires a gradual breaking of hydrogen bonds. In 4‐helix bundles, the termini are at the same end, and applied force acts on the weak hydrophobic contacts, peeling helices away from the bundle 22.

#### Talin dimers

Full‐length talin is dimeric, and helix 62 [dimerisation domain (DD)] forms an antiparallel dimer with another talin molecule 74. In all our experiments to date, we see talin as a constitutive dimer when the DD is present; however, a calpain cleavage site immediately prior to the DD means it can be cleaved to yield monomeric talin 71. Interestingly, the DD in talin2 is conserved with talin1, and structural predictions suggest it should be able to form heterodimers. However, to our knowledge, heterodimers have not been described in the literature. Dimeric full‐length talin1 can adopt a compact autoinhibited conformation in the cytosol 78 where the two rod domains wrap around to form a ‘double donut’ with the two talin heads buried inside. Activation of talins to a more open‐active conformation requires a variety of activators.

## Talin1 and talin2 rod interactions

To date, all the ligands that bind to talin1 have been shown to bind talin2 although the affinities for the different isoforms can be markedly different. Binding partners can interact with the talin rod domains via a number of different modes, that is, to the folded rod domains, to the unfolded rod domains or to some strained conformation between these two extremes. Mechanical force can drive transitions between these conformations and so dramatically alter the binding affinities of different ligands.

### Ligand‐binding sites in the talin rod

Elucidation of the domain structure of the talin rod has enabled the precise mapping of established ligand‐binding sites, and the location of these sites is shown in Fig. 2.

#### Integrin

As well as interacting with the talin head, the beta‐integrin tail also interacts with the R11–R12 domains of the talin rod [integrin binding site 2 (IBS2)] via a structurally undefined mechanism 79, 80, 81. Unlike IBS1 where the integrin binds to the folded F3 subdomain, integrin binding to IBS2 appears to involve some intermediate conformation of the rod domains (integrin does not bind folded or unfolded R11–R12). The role of IBS2 in flies 79, 82 has been well established, but its role in mammals is less clear, although it has been linked to nascent adhesion formation 83.

#### Actin

The talin rod contains two actin‐binding sites, ABS2 (R4–R8) 68, 84, 85 and ABS3 (R13‐DD) 8, 86 which play different roles in adhesion. The current model of talin function envisages the C‐terminal ABS3 74 as responsible for the initial force exerted on talin that leads to unfolding of the mechanosensitive talin rod domain, R3. This triggers vinculin interactions and leads to adhesion maturation 23. In contrast, ABS2, in the centre of the rod provides the tension‐bearing actin connection 84, 85. As with the integrin connections, the actin‐binding sites in talin2 bind more tightly to actin than the equivalent regions in talin1 (35 and our unpublished data).

#### Vinculin

**Figure 3:**
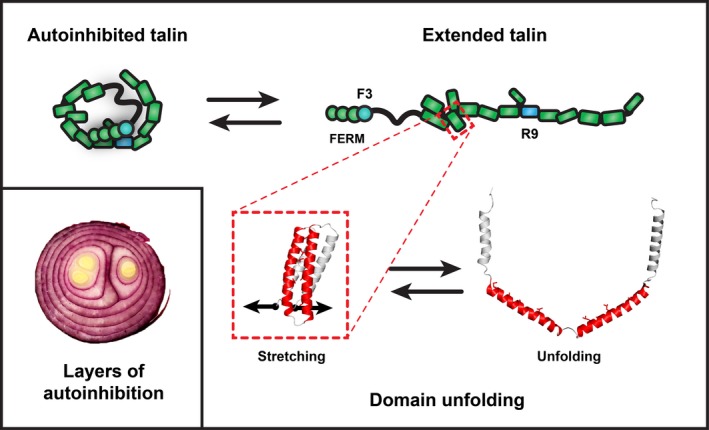
Layers and layers of autoinhibition. A striking feature of talins is their remarkable conformational plasticity that enables different ligands to engage the same platform in different conditions; part of this flexibility emerges from autoinhibition. (Left) In the closed autoinhibited form, all of the domains are folded, and many of the ligand‐binding sites for actin, integrin and vinculin are thought to be cryptic. Some binding sites may face outwards and remain accessible; for instance, RIAM is able to bind to the inactive conformation 127. In the extended conformation in the absence of force, all the domains are still folded, and additional binding sites are exposed (IBS1, IBS2, ABS3, plus the sites for those ligands that require folded‐rod domains) (Right). The exposure of IBS1 and ABS3 facilitates adhesion formation, and by activating integrins and crosslinking them to the actin cytoskeleton, a nascent adhesion can form. As force is exerted on talin, another layer of autoinhibition is uncovered (Bottom). As talin domains unfold, starting with R3, the initial mechanosensor in talin 21, 23, 24, vinculin‐binding sites are exposed and talin:vinculin interactions can now occur. R3 unfolding also reveals the high affinity actin‐binding site in talin, ABS2 that can then activate tension‐bearing actin connections 84, 85. As domains unfold, the binding sites for ligands that engage the folded rod domains are destroyed, as is the case for RIAM binding to R3. A remarkable feature of talins conformational plasticity is that, in the absence of other factors, talin can readily refold to its default low‐force state.

Vinculin, discovered in 1979 87, is another key talin interactor, and has been shown to bind to at least 11 of the 62 talin1 helices 88. The vinculin‐binding determinants lay on one side of each vinculin‐binding helix 89. However, the VBS are buried within the rod domains and are only exposed by mechanical force (Fig. 3), enabling vinculin to bind and strengthen the actin connection. It has been shown that exposed talin VBS can activate vinculin 90, and active vinculin has been shown to be able to activate talin 91. The 11 VBS in talin2 are all conserved and so it is likely that the talin2 rod will also engage vinculin in a similar fashion.

### Other interactors

While the integrin‐, actin‐ and vinculin‐binding interactions define the primary adapter function of talin, there are an increasing number of additional ligands that bind to the talin rod that contribute to its mechanosignalling capabilities. These are summarised below.

#### Talin binds LD‐motif‐containing proteins

A common mechanism for talin rod‐binding proteins is via helix addition, whereby a helix from a ligand packs against the side of a talin rod domain. A number of talin ligands have now been identified that contain an ‘LD‐motif’ 92 that mediates such helix addition. First identified in paxillin 93, amphipathic LD‐motif helices bind via the aspartate (D) which forms an initial salt bridge with a basic residue at the beginning of the furrow between two adjacent helices of the interacting bundle. Specificity is then encoded by residues downstream of the ‘LD’ interaction site. This mode of binding to talin was initially identified from work on the tumour suppressor protein deleted in liver cancer 1 (DLC1) 94, 95. This led on to the identification of the talin‐binding sequence in RIAM 24, 29 as an LD‐motif, and the identification of paxillin as a novel talin ligand 94. More recently, the KANK (kidney ankyrin repeat‐containing) proteins have been identified as LD‐motif‐containing ligands 16, 17, binding to a conserved face on the R7 5‐helix bundle. The ability of 5‐helix bundles to bind LD‐motif proteins greatly expands the number of potential ligand‐binding sites in talin.

#### The talin‐moesin‐NHE‐1 complex and *pH* modulation of adhesion sites

The C‐terminal part of the talin rod has also been shown to bind directly to the FERM domain of moesin, an interaction that is required to recruit the sodium/hydrogen exchanger (NHE‐1) to adhesion sites 96. This recruitment of a proton exchanger to adhesions and the resulting localised alterations of intracellular pH has a dramatic effect on adhesions. Small changes in local pH can result in protonation/deprotonation of side chains, particularly histidines, and this can directly alter interactions in a similar fashion to phosphorylation. In many ways, protonation can be considered a post‐translational modification 97. Many important protein:protein interactions have been shown to be regulated by pH in this way, including the interaction of talin ABS3 with actin 98, and it is likely that local fluctuations in pH will alter the protonation state of many other important interaction sites.

#### The talin–alpha‐synemin connection – a link to intermediate filaments?

Another ligand that has been linked to talin which has the potential to have a significant impact on our view of adhesions is alpha‐synemin 99, an intermediate filament (IF) protein expressed in skeletal muscle. This suggests that talin has the potential to coordinate interactions between the actin, microtubule and IF networks.

## The mechanical properties of talin

The mechanosensing abilities of talin rely on its force‐dependent interactions with its binding partners. Some ligands (i.e. RIAM, KANK, DLC1, actin) bind only to folded talin domains, whereas vinculin is known to require domain unfolding and exposure of cryptic VBS. Force‐induced talin domain unfolding will therefore release binding partners that bind to folded talin and stimulate binding of vinculin, triggering mechanosensing signals. *In vivo*, talin is initially extended by actin retrograde flow and then by actomyosin contractility and the resulting forces exerted on the talin rod drive structural transitions. Depending on the precise mechanical environment, individual talin molecules will experience different forces, and the different conformations may engage different ligands.

### Mechanotransduction: force driving changes in biological signalling

The mutually exclusive interactions between talin and RIAM and talin and vinculin provide the perfect example of how talin can convert mechanical forces into biological signalling responses. The initial mechanosensitive domain in talin has been shown to be R3 23, 24, which binds RIAM but also contains two VBS. However, vinculin and RIAM have fundamentally different modes of binding. Talin VBS are buried within the talin rod domains and are only exposed when mechanical force unfolds that domain, allowing vinculin to bind. In contrast, the talin‐binding sites in RIAM are single helices that interact only with folded talin rod domains (Fig. 2). The exquisite mechanosensitivity of R3 is due to the presence of a destabilising cluster of threonine residues buried in its hydrophobic core 24. This means that the R3 domain is the first to unfold when talin experiences force, driving the transition between folded and unfolded R3 (this is one of the exciting aspects of structural mechanobiology in that the precise structural basis of a mechanosensitive event can be pinpointed to specific amino acids that encode the mechanosensitivity). This conformational change in R3 drives a change in biological signalling, displacing RIAM and thus the link to the Rap1 signalling pathways. Simultaneously, R3 unfolding leads to the recruitment of vinculin and strengthening of the connection to actin. This allows two different ligands to engage the same talin domain under different conditions and explains the different localisation of RIAM and vinculin in cells 100. In the case of R3, a force of ~ 5 pN is required for it to unfold, disrupting the RIAM‐binding sites and recruiting vinculin, driving the maturation of nascent adhesions into FAs. This 5 pN force is roughly the force of a single actomyosin contraction, leading to an attractive hypothesis that talin only experiences this force threshold when it binds to an integrin and simultaneously connects to the actin cytoskeleton. Only when these two criteria are met will the R3 domain unfold and trigger adhesion maturation.

Expanding this to the rest of the talin rod, it seems likely that each of the talin rod domains can also serve as mechanochemical switches, and under different conditions, individual talin rod domains can adopt different conformations that support different signalling pathways. *In vivo* measurements of talin extension have shown that talin length is normally between 90 and 250 nm 101 (compared with a folded talin length of 50–60 nm *in vitro* 102), suggesting that between two and eight talin rod domains are unfolded at any time 21. Using single molecule analysis with ultrastable magnetic tweezers, we recently characterised the mechanical response of talin1 21, 22, 23. Stretching the whole talin rod revealed quantised mechanical responses with all 13 rod domains exhibiting switch‐like behaviour at different force thresholds. These unfolding responses range from ~ 5 to 25 pN and are all rapidly reversible when force is removed. This reveals a spectrum of mechanosensitive switching events, turning on and off distinct effector functions in a force‐dependent manner. This stochastic force‐dependent folding and refolding of talin also make talin an effective force buffer protecting adhesions against excessive force 21.

The R8 domain is a hotspot for protein interactions, suggesting it represents a major signalling hub. R8 is a 4‐helix bundle, uniquely protected from mechanical force by being inserted into the loop of a 5‐helix bundle (R7), creating a novel 9‐helix module, and a branch in the talin rod 21, 75 (Fig. 2). By being positioned outside of the force‐bearing region, R8 remains folded whilst talin is under force and maintains its ligand‐binding surface.

A striking feature of the talin rod's response to force is that even after complete unfolding, the removal of force leads to refolding to the original native state, and this response is maintained through multiple cycles of extension and relaxation. The robustness of the mechanical response of the talin rod is perfectly suited to its role as a mechanosensor; when the mechanical force is relieved, the sensor reverts back to its original state.

Together, these features suggest that talins can sense and respond to mechanical forces with remarkable versatility. Depending on the applied force, different domains will unfold, and depending on the repertoire of expressed ligands, different signals will be generated. Depending on the mechanism of linkage to actin (e.g. via ABS2 vs. ABS3), or to microtubules via KANK, different regions of talin will be under tension. This network of protein interactions thus provides a mechanism for context and force‐dependent regulation of multiple signalling pathways.

It will be important to characterise the mechanical response of talin2 as differences in mechanical responses of individual rod domains might help pinpoint sites of functional divergence. Recent work has shown that the two talins provide different mechanical linkages in cells 34, with talin2 able to engage vinculin in the absence of mechanical force, suggesting that the two proteins respond to forces differently. Talin2 is expressed at high levels in cardiac and skeletal muscle 103 where presumably its higher affinity for integrin beta1d and actin may serve to create more resilient adhesive connections.

### Talin: layers and layers of autoinhibition

An interesting feature of talins is that the binding sites described above are not all accessible all of the time. Talin activity is regulated by multiple layers of autoinhibition where binding sites are masked, and only made available for binding in response to different signals. Talin autoinhibition mediated via the interaction between the integrin‐binding site in F3 and the talin rod domain R9 maintains talin in a compact cytosolic form 104, 105, 106, 107. The F3‐binding surface on talin2 R9 is highly conserved with only subtle conservative changes, and as such autoinhibition is likely common to both talins. Multiple factors (e.g. PIP2, FAK, Vinculin, RIAM, etc.) have been implicated in relieving talin autoinhibition, most recently, the G‐protein Gα13 which binds F3, displacing the R9 rod domain, has emerged as an important talin regulator 108. Once autoinhibition is relieved, it is likely that some of talins functionalities are exposed, such as the integrin‐ and membrane‐binding sites on the talin head 52, 109, and the C‐terminal actin‐binding site ABS3 8, 74. However, other functions are still autoinhibited; for example, the VBS remain inaccessible, buried in the hydrophobic core of the rod domains. As mechanical force is exerted on talin, its rod domains can unfold, exposing VBS and simultaneously destroying the binding sites for folded rod binders, enabling mechanochemical switching of binding. In this scenario, once RIAM has served its purpose and helped translocate talin to the plasma membrane, its binding to talin is no longer required and so those domains are repurposed for alternative functions. Furthermore, high affinity actin binding is mediated via the central actin‐binding site (ABS2; R4–R8) which is maintained in an inactive conformation via the inhibitory effects of the adjacent R3 and R9 domains 84. As a result of this stratified nature of talin autoinhibition, the same protein scaffold can coordinate many different processes. There are likely numerous other talin functions tightly regulated by talin conformation in a similar fashion.

### Comparison of the talin1 and talin2 domains

Due to the high homology between talin1 and talin2, we used Modeller 110 to generate structural models of the talin2 domains using the talin1 structures as templates. Validation of the conserved hydrophobic cores of these domains and comparison of the modelled F2F3 region of talin2 with the known structure 52 confirmed the reliability of this modelling approach. The domain boundaries of talin1 and talin2 are shown in Fig. 2. From this bioinformatics analysis, we have designed and validated expression constructs to express and purify each of the talin2 domains (deposited in Addgene http://www.addgene.org/ben_goult). We have recently solved the structures of a number of these talin2 rod domains and it is striking how structurally similar they are to the equivalent talin1 domains (our unpublished data).

### Conserved differences between the two isoforms

The two talins are highly conserved (76% identical), and it is likely that identical regions between the two isoforms carry out equivalent functions. What has not been explored in detail is the 24% of the sequence that is not identical – it is here that differences in isoform function might be found. In particular, we sought to identify conserved differences between the isoforms in these divergent regions as these might provide the key to understand the differences in isoform function. We set out to look at differences in ligand specificity, affinity tuning, tertiary structure and the conservation of post‐translational modification sites (PTMs).

We used BlastP to align the sequences of the corresponding domains from each isoform to establish the identity and similarity of each domain and to look for local variations. Interestingly, although the sequence identity between the two talins is 76%, sequence identity at the domain level shows much greater variation. The F2 (86%), F3 (89%) and R13 (92%) are highly conserved between isoforms, supporting their role in assembly of the core adhesion complex. In contrast, other regions of the talin rod show considerable variation in conservation between isoforms with R5 (60%) being the most divergent.

Using a sample set of vertebrates, we compared the conservation of each individual talin domain between species and between isoforms. Regions where sequence conservation is low within and between the individual isoforms likely represent regions of less functional importance. In contrast, regions that are highly conserved within an isoform but are less conserved between isoforms might indicate regions of functional divergence. This structure‐oriented conservation analysis reveals that for some talin rod domains, the binding surfaces are completely conserved between isoforms. For example, talin rod domains, R7 and R8, both contain binding sites for LD‐motif‐containing proteins, and the R7‐ and R8‐binding surfaces on both talin isoforms are identical. This is reflected by the similar binding constants (*K*_d_) of these domains for their respective ligands, that is, the KANK 1 and KANK 2 binding site on R7 and the RIAM‐ and DLC1‐binding site on R8. These proteins bind in the same manner and with the same affinity to both isoforms (16 and our own unpublished data).

In contrast, the R5 domain, for which ligands have yet to be identified, has a highly conserved surface in talin2 with the characteristics of an LD‐motif‐binding domain, but this surface is markedly different in talin1. Based on this analysis, we suggest that regions of divergence between isoforms that are well conserved within each isoform likely encode regions that define the subtle differences in isoform functionalities.

## Talin2 in disease and development

The roles of talin2 during embryogenesis and development are not fully understood, but studies of the two talin isoforms in the heart reveal that they are tightly regulated 5, 111. Both isoforms are highly expressed in cardiomyocytes, but during maturation, and in the mature heart, talin2 becomes the major isoform, localising to the costameres 111. Indeed, cardiac‐specific talin1 knockout mice show normal basal cardiac function. Interestingly, talin1 is upregulated in the failing human heart, and studies in mice show that an ablation of cardiac talin1 blunts the hypertrophic response and improves cardiac function 106. The mechanisms behind isoform switching in heart remain to be elucidated, but the data clearly indicate that the two talin isoforms play distinct roles in cardiac muscle. Further evidence of the importance of talin2 in development comes from the exome sequencing‐based identification of a mutation (S339L in F3) in the *Tln2* gene that causes fifth finger Camptodactyly 112. Given that talin2 is not an essential gene, it seems likely that whole exome sequencing will reveal further disease‐associated mutations in the *Tln2* gene, and these will provide further insights into its functions.

Interestingly, the *Tln2* gene also includes a highly conserved microRNA, miR‐190, situated in intron 51 10, which has been implicated as a modulator in multiple signalling pathways. Moreover, talin2 has appeared in a number of screens as a protein regulated by microRNAs whose expression is perturbed in cancer 53, 113. Thus, the humanised antibody trastuzumab, which recognises the extracellular domain of HER2, upregulates miR‐194 expression in two HER2‐positive breast cancer cell lines 113, and miR‐194 suppresses cell migration reportedly via downregulation of talin2. Talin2 is also downregulated by miR‐132, but miR‐132 expression is itself suppressed by promoter methylation in prostate cancer cells. This correlates with a worse prognosis, and the authors speculate that elevated talin2 levels may suppress cell death and increase metastasis 114. Talin2 upregulation has also been implicated in breast cancer tumorigenesis and metastasis 33, 53 driving more aggressive cell invasion.

## Post‐translational modification of talins

Talin has been shown to be regulated by post‐translational modification and the phosphorylation sites in platelet talin1 have been mapped 115. Proteomics studies on the ‘adhesome’ also show phosphorylation of talin in adhesions 116, but there is much less data on talin2 PTMs. To explore this, we took all reported phosphorylation and acetylation sites for talin1 and found that the majority were conserved in talin2 (Table 1). Mass spectrometric analysis of calyculin‐treated platelets identified numerous talin1 phosphorylation sites, with the three most abundant sites being T144 and T150 in the F1‐loop (phosphorylation appears to negatively regulate integrin activation 58, 115) and S446 115 in the linker between the head and rod domains. Phosphorylation of S446 is believed to be important in the regulation of the calpain cleavage between the head and the rod domain and is important for regulating FA turnover 71, 117, a process that has recently been shown to be essential for adhesion development and rigidity sensing 118. In addition, S425, which is also in the linker, is phosphorylated by CDK5, and this phosphorylation has been shown to enhance talin activity and increase integrin activation 119.

**Table 1:** Post‐translational modifications in talin1 and talin2. Summary of the identified talin phosphorylation 115, 116, acetylation 128, arginylation 121, glycosylation 123 and methylation sites 122. For each PTM, the modified residue, the domain it is located, and the conservation between isoforms are shown. Residue numbering is for mouse talin1 and talin2

Talin1 phosphorylation site	Domain of talin1	Site conserved in talin2	Talin1 phosphorylation site	Domain of talin1	Site conserved in talin2
S5	F0	Yes	S677	R2	No
Y26	F0	Yes	S729	R2	Yes
Y70	F0	Yes	S815	R3	Yes
T78	F0	No	S940	R4	No
T96	F1	No	S979/S981	R4	No/Yes
T114	F1	Yes	S1021	R4	Yes
Y127	F1	Yes	Y1116	R5	Yes
S128	F1	Yes	T1142	R5	Yes
T144	F1	Yes	S1201	R5	No
T150	F1	Yes	S1225	R6	No
T167	F1	Yes	T1263	R6	No
T190	F1	Yes	S1323	R6	Yes
S311	F3	Yes	S1508	R8	No
S405	LINKER	Yes	S1641	R7	Yes
S425	LINKER	Yes	S1684	R9	Yes
S429/T430	LINKER	Yes	S1849	R10	No
Y436	LINKER	No	T1855	R10	Yes
S446	LINKER	Yes	S1878	R10	No
S455/S458	LINKER	Yes	S2040	R11	No
S467	LINKER	Yes	S2127	R11	Yes
S620	R1	Yes	S2338	R12	No
			Y2530	DD	Yes
			S2535	DD	No

Talin2 phosphorylation site	Domain of talin2	Site conserved in talin1	Talin1 PTM	Domain of talin1	Site conserved in talin2
Y1665	R9	No	K1544 (acetylation)	R8	Yes
T1843	R10	No	K2031 (acetylation)	R11	Yes
			K2115 (acetylation)	R11	Yes
			A1903 (arginylation)	R10	Yes
			T1487 (glycosylation)	R8	No
			T1890 (glycosylation)	R10	Yes
			K2454 (methylation)	R13	Yes

Calpain cleavage of talin is a permanent PTM, and three cleavage sites have been identified in talin1. The best characterised is that within the linker between the talin head and rod (between residues Q433 and Q434) 73, but there is a second site immediately prior to the DD (between residues K2493 and M2494) 71. Both of these cleavage sites are present in talin2 71. Calpain cleavage of the neck exposes a recognition site for the E3 ligase SMURF1 that leads to ubiquitination of the liberated talin1 head 120. A third force‐dependent calpain cleavage site in the talin1 rod has also been identified 121. This cleavage occurs between residue P1902 and A1903 (a site which is normally buried in the folded R10 domain) and is likely to only be accessible when talin is under force. This cleavage appears to be regulated by arginylation (a PTM that only occurs on the N‐term residue of proteins 121). It is not yet known whether this force‐dependent cleavage site is also present in talin2, but the region is well conserved between both isoforms.

Finally, a number of additional PTMs have also been identified in talin1. For instance, the affinity of the talin1:actin connection is controlled via the methyltransferase Ezh2 which methylates talin at lysine K2454 in ABS3 122. This PTM site is completely conserved in talin2. Talin1 is also modified by glycosylation 123 at sites in R8 and R10. Interestingly, the glycosylation sites are not conserved between talin1 and talin2, suggesting that if talin2 is glycosylated, then it is at different sites and linked to different functions.

## Conclusions and perspectives

Gene duplication is often viewed as an evolutionarily advantageous process, with duplicated genes giving rise to two proteins that can acquire distinct or completely new functions (subfunctionalisation or neofunctionalisation). Gene duplication may also allow more complex patterns of gene expression in different cell types and tissues 124. Furthermore, differences in PTM sites as reported here for the two talins may enable new modes of regulation at the protein level. Although both talin isoforms have maintained their ancestral properties relating to cell adhesion, it seems likely that the two isoforms have undergone some neofunctionalisation to generate nuanced, isoform‐specific regulation of signalling in cell adhesion. We imagine a scenario whereby talin2 plays a central role in some tissues, such as cardiac muscle, the brain and kidney, but then also a more global role in fine‐tuning the adhesive response in many other cell types. Recent work has shown that the two talins provide different mechanical linkages in cells 34, and the ability to measure talins mechanical response both at the single molecule level 21, in cells using genetically encoded tension sensors 34, 85, 125, and *in silico* with force extension molecular dynamics simulations 126 provide the tools to understand how talin signalling varies with mechanical forces. Detailed structural and biochemical characterisation of talin interactions is enabling targeted mutations to be designed that specifically disrupt individual talin functions, which in conjunction with the aforementioned technical advances should enable the study of talin function in unprecedented detail. It is likely that further novel talin‐mediated cell functions will be identified as additional binding partners of the two talins are discovered.

Many different adhesive structures form on talins which signal in a highly reproducible manner. Precisely how talins’ mechanosignalling capabilities are integrated with the more classical signalling pathways to give rise to these robust metastable cellular responses that facilitate all our cellular processes remain to be determined. The signalling pathways that regulate talin function, localisation, post‐translational modifications, etc. coupled with the forces that define the conformational status of its rod domains, which cumulatively lead to the correct cellular responses are still poorly understood. For talin to generate robust, reproducible signalling responses and specialised adhesive structures in response to such diverse, multiple inputs suggest that there must be a code underpinning talins mechanotransductive response. Deciphering this ‘talin code’ is the next major challenge.
